# 4052 A TL1 Team Approach to Personalization of Donor Human Milk for Preterm Infants

**DOI:** 10.1017/cts.2020.286

**Published:** 2020-07-29

**Authors:** Natalie Harrison, Marion M Bendixen, Josef Neu, Leslie A. Parker, Graciela L. Lorca

**Affiliations:** 1 University of Florida Clinical and Translational Science Institute

## Abstract

OBJECTIVES/GOALS: Feeding preterm infants with mother’s own milk (MOM) lowers rates of sepsis, decreases necrotizing enterocolitis, and shortens hospital stay. Our objective is to determine whether a similar microbial diversity to MOM can be obtained when fresh or frozen MOM is inoculated in donor human milk (DHM). METHODS/STUDY POPULATION: Subjects included 12 mothers of infants born 100ml of MOM per day and were excluded if they had taken antibiotics within 3 days of the 1-time pumped MOM sample collection. MOM sample was divided into fresh (processed immediately) and frozen (−20°C) for 24h fractions. MOM was inoculated in DHM [referred to as refaunated milk (RM)] at 10% (RM10) and 30% (RM30) dilutions, then incubated at timepoints: 0h, 2h, 4h at 37°C. At each timepoint, total viable microbial cell counts were performed in differential or selective media along with future 16S rRNA sequencing. RESULTS/ANTICIPATED RESULTS: Microbiota expansion was detected in MOM, RM10 and RM30 over time whether fresh or frozen milk was used as the inoculum. Incubated fresh and frozen MOM had similar bacterial loads when tested on nutrient agar (10^5-10^6 CFU/mL), mannitol salt (10^6 CFU/mL), MacConkey (10^2-10^5 CFU/mL), blood agar (10^6 CFU/mL) and MRS (10^4 CFU/mL) plates. Based on these CFU counts, RM30 incubated for 2h and RM10 at 4h showed similar counts to that of MOM at 0h. DISCUSSION/SIGNIFICANCE OF IMPACT: RM, inoculated with fresh or frozen MOM, obtained a similar microbial count compared to MOM at 0h indicates that fresh or frozen MOM can inoculate DHM. 16s rRNA sequencing is ongoing. Future studies are needed to support an inoculation protocol to be used in clinical practice and human milk banking.

